# Epithelial-to-mesenchymal transition in FHC-silenced cells: the role of CXCR4/CXCL12 axis

**DOI:** 10.1186/s13046-017-0571-8

**Published:** 2017-08-03

**Authors:** I. Aversa, F. Zolea, C. Ieranò, S. Bulotta, A. M. Trotta, M. C. Faniello, C. De Marco, D. Malanga, F. Biamonte, G. Viglietto, G. Cuda, S. Scala, F. Costanzo

**Affiliations:** 1 Research Center of Advanced Biochemistry and Molecular Biology, Department of Experimental and Clinical Medicine, Magna Græcia University of Catanzaro, Salvatore Venuta Campus, Viale Europa, 88100 Catanzaro, Italy; 2 Genomica Funzionale, INT Pascale, Napoli, Italy; 3 Department of Health Sciences, Magna Græcia University of Catanzaro, Salvatore Venuta Campus, Viale Europa, 88100 Catanzaro, Italy; 4 Department of Experimental and Clinical Medicine University of Catanzaro “Magna Graecia”, 88100 Catanzaro, Italy

**Keywords:** Ferritin heavy chain, EMT, CXCR4, CXCL12, Cancer, ROS

## Abstract

**Background:**

Ferritin plays a central role in the intracellular iron metabolism; the molecule is a nanocage of 24 subunits of the heavy and light types. The heavy subunit (FHC) is provided of a ferroxidase activity and thus performs the key transformation of iron in a non-toxic form. Recently, it has been shown that FHC is also involved in additional not iron-related critical pathways including, among the others, p53 regulation, modulation of oncomiRNAs expression and chemokine signalling. Epithelial to mesenchymal transition (EMT) is a cellular mechanism by which the cell acquires a fibroblast-like phenotype along with a decreased adhesion and augmented motility. In this work we have focused our attention on the role of the FHC on EMT induction in the human cell lines MCF-7 and H460 to elucidate the underlying molecular mechanisms.

**Methods:**

Targeted silencing of the FHC was performed by lentiviral-driven shRNA strategy. Reconstitution of the FHC gene product was obtained by full length FHC cDNA transfection with Lipofectamine 2000. MTT and cell count assays were used to evaluate cell viability and proliferation; cell migration capability was assayed by the wound-healing assay and transwell strategy. Quantification of the CXCR4 surface expression was performed by flow cytometry.

**Results:**

Experimental data indicated that FHC-silenced MCF-7 and H460 cells (MCF-7^shFHC^, H460^shFHC^) acquire a mesenchymal phenotype, accompanied by a significant enhancement of their migratory and proliferative capacity. This shift is coupled to an increase in ROS production and by an activation of the CXCR4/CXCL12 signalling pathway. We present experimental data indicating that the cytosolic increase in ROS levels is responsible for the enhanced proliferation of FHC-silenced cells, while the higher migration rate is attributable to a dysregulation of the CXCR4/CXCL12 axis.

**Conclusions:**

Our findings indicate that induction of EMT, increased migration and survival depend, in MCF-7 and H460 cells, on the release of FHC control on two pathways, namely the iron/ROS metabolism and CXCR4/CXCL12 axis. Besides constituting a further confirmation of the multifunctional nature of FHC, this data also suggest that the analysis of FHC amount/function might be an important additional tool to predict tumor aggressiveness.

## Background

Epithelial to mesenchymal transition (EMT) is a rapid and often reversible phenomenon characterized by morphological changes of epithelial cells. The activation of the EMT process leads to cytoskeleton re-organization, loss of cell-cell adhesion molecules, modification of cellular polarization, de novo expression of mesenchymal proteins and acquisition of stemness properties [1, 2]. Thus, the cells undergoing EMT gain increased cell motility, invasive properties and resistance to anoikis [3, 4]. EMT plays a role in normal development, such as heart morphogenesis and neural crest formation [5]. However, the acquisition of a mesenchymal phenotype makes cancer cells more aggressive and metastatic, therefore suggesting that EMT might be considered as an attractive therapeutic target [6–8].

A variety of studies performed both in vitro and in vivo have focused on the identification of EMT-inducing agents, underscoring, among others, the role played by the reactive species of oxygen (ROS), hypoxia and perturbations of the intracellular iron metabolism [9, 10]. Ferritin, a globular protein of 450 kDa, is the major iron storage protein within the cell, thus playing a central role in the maintenance of redox homeostasis [11]. Ferritin is present in cytoplasm, nucleus and mitochondria; the cytoplasmic form is composed by 24 subunits of heavy (FTH, FHC) and light type (FTL), assembled to form a central cavity where the iron atoms are stored [11]. FHC and FTL share an extensive homology in the sequence but perform different functions in iron metabolism: FHC holds a ferroxidase activity and is devoted to rapid iron uptake and release, while FTL contributes to the long-term iron storage [12]. The enzymatic activity held by FHC makes it a key molecule in the control of the intracellular free iron and, as a consequence, of ROS production; therefore it has been hypothesized, and demonstrated in given cell types, a role for FHC in EMT induction [10].

Besides iron metabolism, FHC controls additional biochemical pathways such as cell proliferation [13], p53 regulation [14], chemokine signalling [15], angiogenesis [16], regulation of oncomiRNAs network [17], control of proper protein folding [18], stem cell expansion [19]. In some pathways FHC physically interacts with target molecules independently from its ferroxidase activity [19]. Among the molecules whose activity is modulated upon FHC binding there is the chemokine receptor CXCR4 [15]. CXCR4 is a seven-span transmembrane G protein-coupled receptor (GPCR) that binds the Stromal-Derived Factor-1 (SDF-1 or CXCL12) ligand [20]. CXCR4 is widely expressed in different tissues, where it plays a central role in development-related processes; deficit of the CXCR4/CXCL12 axis determines severe alterations of the hematopoietic, immune and CNS compartments [21]. Moreover, CXCR4 expression is increased in a variety of human cancers [22–25] where it regulates tumor progression and EMT [26–28].

In this paper we provide evidence that FHC silencing in the human breast cancer MCF-7 and in the lung adenocarcinoma H460 cells induces EMT and enhances proliferative and migratory abilities. In these cell lines, FHC knock down is accompanied by an increase in ROS content and by an approximately two-fold increase of CXCR4 receptor expression, along with a strong activation of its downstream pathways. ROS increase largely contributes to the higher proliferation rate of FHC-silenced cells, while their enhanced migratory ability is mainly ascribable to the activated CXCR4/CXCL12 axis.

The data presented here add new insights into the role of FHC in transformed cells, indicating that perturbations of its intracellular content are accompanied by an up-regulation of the CXCR4/CXCL12 axis, which, in turn, contributes to the acquisition of a more aggressive phenotype.

## Methods

### Cell cultures

MCF-7 human breast adenocarcinoma cells (ATCC, Manassas, VA, USA) were cultured in DMEM (Sigma-Aldrich, St. Louis, MO, USA) medium supplemented with 10% fetal bovine serum (FBS) and 1% Penicillin-Streptomycin (Sigma-Aldrich); H460 human non-small lung cancer cell (ATCC) were cultured in RPMI 1640 (Sigma-Aldrich) medium supplemented with 10% fetal bovine serum (FBS) and 1% Penicillin-Streptomycin (Sigma-Aldrich). The two cell lines were maintained at 37 °C in a humidified 5% CO2 atmosphere. HEK-293 T cells (Sigma-Aldrich) were cultured in adherent conditions in DMEM (Sigma-Aldrich) medium with 10% FBS and 1% Penicillin-Streptomycin at 37 °C in a humidified 5% CO2 atmosphere.

### Preparation of lentiviral supernatants and transduction of MCF-7 and H460 cells

Lentiviral preparation and transduction were performed as described in Di Sanzo et al. [29]. Cells were stably transduced with a lentiviral DNA containing either an shRNA that targets the 196–210 region of the FHC mRNA (sh29432) (MCF-7^shFHC^) (H460^shFHC^) or a control shRNA without significant homology to known human mRNAs (MCF-7^shRNA^) (H460^shRNA^). A pool of clones were analysed after puromycin selection.

### FHC reconstitution

MCF-7^shFHC^ cells were seeded in six-well plates at 6 × 10^5^cells/well and grown overnight prior to transfection. All plasmids were transfected with Lipofectamine 2000 transfection reagent (Thermo Fisher Scientific, Waltham, MA, USA) following manufacturer’s instructions.

FHC reconstitution was performed using 2 μg/μl of the expression vector containing the full length of human FHC cDNA (pcDNA_3_/FHC) (MCF-7^shFHC/pcDNA^_3_^FHC^) while 2 μg/μl of pcDNA_3_ plasmid was used as negative control (MCF-7^shFHC/pcDNA^_3_). Transfection efficiency was tested 48 h. All transfection experiments were repeated in triplicate.

### RNA extraction and semi-quantitative reverse transcriptase polymerase chain reaction (RT-PCR)

Total RNA was extracted from FHC-silenced MCF-7 and H460 (MCF-7^shFHC^, H460^shFHC^) and scrambled-shRNA MCF-7 and H460 (MCF-7^shRNA^, H460^shRNA^) by using Trizol method according to the manufacturer’s instructions (Thermo Fisher Scientific). All the RNA samples were DNase-1 treated (Ambion, Austin, TX, USA), and purity and integrity of the RNA was checked spectroscopically and by gel electrophoresis before use. Then, 1 μg of purified RNA was reverse-transcribed by using High capacity cDNA Reverse Transcription kit (Thermo Fisher Scientific) [30].

### Western blotting analysis

MCF-7 and H460 silenced and un-silenced cells were lysed in the following buffer (20 mM Hepes pH 7.9, 420 mM NaCl, 1% Triton ×-100, 1 mM EDTA, 25% glycerol, 1 mM PMSF, 1 mM Na_3_VO_4_, 1 mM DTT, 1 μg/ml aprotinin, 1 μg/ml leupeptin) for 30 min on ice. After removing cell debris by centrifugation (12,000 g × 30 min), the concentration of protein extracts was measured by the Bio-Rad protein assay according to the manufacturer’s instructions (BioRad, California, USA). Total protein extract was boiled for 10 min in SDS sample buffer, separated by 10–12% SDS-PAGE and transferred to a nitrocellulose membrane by electroblotting. Non-specific reactivity was blocked in nonfat dry milk in TPBS [5% (*w*/*v*) milk in PBS (pH 7.4) and 0.005% Tween 20] for 2 h at room temperature. The membrane was incubated with rabbit anti-H ferritin (1:200; Santa Cruz Biotechnology, Texas, USA), rabbit anti-CXCR4 antibody (1:500; Abcam, Cambridge, UK), rabbit anti-VIMENTIN, anti-E-CADHERIN antibodies, anti-phospho-AKT (Ser473), anti-AKT, anti-P70S6 K, anti-phospho-P70S6 K (T389) (1:1000; Cell Signaling Technology, Danvers, MA, USA) over-night at 4 °C, followed by incubation with goat anti-rabbit secondary antibody (1:5000; Santa Cruz Biotechnology). The membrane was developed by ECL-Western blot detection reagents according to the manufacturer’s instructions (Santa Cruz Biotechnology). γ-Tubulin was used as a loading control.

### Quantitative real-time PCR (qRT-PCR)

Gene expression analysis was assessed by real-time PCR using the cDNA obtained from MCF-7^shRNA^, MCF-7^shFHC^, MCF-7^shFHC/pcDNA^_3_^FHC^ and from H460^shRNA^ and H460^shFHC^ cells.

Primer sequences used for amplifications were as follows:

GAPDH-F 5′-aac acc acc atg gag aag gc −3′; GAPDH-R 5′-aca gcc ttg gca gca cca ct-3′;

FHC FW: 5′-cat caa ccg cca gat caa c-3′; FHC REV: 5′-gat ggc ttt cac ctg ctc at-3′.

Twist-F 5′-tga atc ttg ctc agc ttg tc-3′; Twist-R 5′-cgg gcg tcc gga gtc tta-3′;

Slug-F 5′-ggt gtc aga tgg agg agg g-3′; Slug-R 5′-cat gcc tgt cat acc aca ac-3′;

Vim-F 5′-agg aaa tgg ctc gtc acc ttc gtg-3′ Vim-R 5′-gga gtg tcg gtt gtt aag aac tag-3′;

E-cadherin-F 5′-tac gcc ggg act cca cct a-3′; E-cadherin-R 5′-cca gaa agc gag gcc tga t-3′.

20 ng of cDNA was amplified in 20 μl of reaction mix containing Power SYBR Green PCR Master mix (Thermo Fisher Scientific), 20 pmol of each primer pair and nuclease-free water. The thermal profile consisted of 1 cycle at 95 °C for 10 min followed by 40 cycles at 95 °C for 15 s, 60 °C for 1 min. The human GAPDH cDNA fragment was amplified as the internal control. Data analysis was performed using the 2^-ΔΔCt^ [31].

### Immunofluorescence

MCF-7 and H460 cells were cultured on cover slip. After two wash in PBS, cells were fixed with 4% paraformaldehyde (PFA) (Sigma Aldrich) in Sodium Posphate solution 0.120 M pH = 7.4, for 20 min at 37 °C and then washed in PBS. Cells were washed twice with high salt solution (HS: NaCl 500 mM, Na_3_PO_4_ pH = 7.4 20 mM) and then permeabilized in HS buffer containing Triton-X-100 0.3% and jelly at 0.2% for 30 min at room temperature in a humidified room. To stain actin filaments, cells were incubated for 30 min in this buffer containing Oregon Green® 488 phalloidin at 1:400 dilution (Molecular Probes, Thermo Fisher Scientific), while primary antibodies (ferritin heavy chain, 1:100 Santa Cruz Biotechnology; Vimentin and E-Cadherin, 1:100, Cell Signaling) were diluted in the same buffer for 2 h at room temperature. Appropriate secondary antibodies (antimouse IgG Alexa Fluor 488 and anti-rabbit IgG Alexa Fluor 555, Thermo Fisher Scientific) diluted in HS buffer were applied for 1 h at room temperature. After 3 washes with HS buffer and 1 wash with PBS, Nuclear Dapi (1:1000, Invitrogen, Carlsbad, CA) was added for 20 min (ProLong Gold antifade Reagent, Molecular Probes, Eugene, OR). The slides were mounted on microscope slides using a mounting solution ProLong Gold antifade reagent (Thermo Fisher Scientific). Images were collected using a Leica DM-IRB/TC-SP2 confocal microscopy system (40×, 63× or 100× objective) at 1024 × 1024 resolution pixel.

### ROS detection

ROS were determined by incubating MCF-7 and H460 silenced and unsilenced cells with the redox-sensitive probe 2′-7′-DCF (CM-H2CFDA; Molecular Probes, Eugene, OR) [32]. Briefly, 1 × 10^6^ cells were plated in 96-well plates and incubated with Hanks balanced saline solution (HBSS), 10 mM glucose and 20 μm DCF for 15 min at 37 °C. After two cycle washes, cells were maintained in HBSS supplemented with 10 mM glucose. Fluorescence was measured using the FACS FORTESSA.

### Cell viability and proliferation assays

Cell viability was tested using a 3-(4,5-dimethylthiazol-2-yl)-2, 5-diphenyl tetrazolium bromide (MTT) (Sigma-Aldrich) assay. For the assay, 3 × 10^3^ FHC-silenced and unsilenced MCF-7 and H460 cells were plated in 96-well flat bottom tissue culture plate. At 24, 48 and 72 h of culture, 10 μl of MTT solution (2 mg/mL) were added per well. After 6 h of incubation crystals of formazan were solubilized with 50 μl of 2-Propanol (Sigma-Aldrich). Optical density (O.D.) was read on a multiwell scanning spectrophotometer (ELISA reader) at 450 and 595 nm.

To assess cell proliferation MCF-7 and H460 cells were plated in DMEM and RPMI complete media. Cell counts using trypan blue were performed at 0, 24, 48 and 72 h.

### Glucose and lactate assay

MCF-7 and H460 cells (1 × 10^5^) were seeded in 10 ml of DMEM (MCF-7) or RPMI 1640 (H460) (Sigma-Aldrich), supplemented with FBS and Penicillin-Streptomycin (Sigma-Aldrich) in 100 mm plates. After 24, 48, 72 and 96 h, 500 μl of supernatant were taken and glucose and lactate concentration was measured using COBAS 6000 instrument (Roche, Indianapolis, IN, USA). The assay was performed three times.

### Wound healing assay

MCF-7 and H460 cells were seeded in a 6-well plates and cultured until reaching confluence*.* For simulating a wound, a (yellow) pipette tip was used to make a scratch. At 0, 24 and 48 h, cells were monitored and images of wound healing were captured (magnification of 10X) using the Leica DFC420 C and Leica Application Suite Software. Subsequently, cell migration was quantified by measuring the wound opening area with ImageJ64 software.

### Quantification of CXCR4 surface expression

MCF-7 and H460 cells (1 × 10^6^) were harvested and rinsed twice, and 1% bovine serum albumin (BSA) in PBS solution was used to block the cells for 30 min in an ice bath. Then cells were stained with anti-CXCR4 PE-antibody (FAB170P, clone 12G5, R&D Systems, Minneapolis, MN, USA) for 1 h at 4 °C. After antibody staining, cells were rinsed with 1% BSA in PBS three times, resuspended in PBS, and evaluated by a FACS Canto II cytofluorometer (Becton Dickinson Immunocytometry Systems, Mountain View, CA, USA).

### Migration assay

Migration was assayed in 24 transwell chambers (Corning Inc., Corning, NY, USA) using inserts with 8-μm pore membrane. MCF-7 and H460 cells were placed in the upper chamber (2 × 10^5^cells/well) in DMEM containing 0.5% BSA (migration media) plus/minus AMD3100. CXCL12 (100 ng/mL) was added to the lower chamber. After 18 h of incubation, cells on the upper surface of the filter were removed using a cotton wool swab; the cells that had migrated onto the lower surface of the membrane were stained with DAPI, photographed and visually counted in 10 random fields. Migration index is the ratio between number of migrated cells / number of migrating cells toward CXCL12 free media [33].

### cAMP assay

MCF-7^shRNA^ and MCF-7^shFHC^ cells were pre-incubated for 30 min at 37 °C with AMD3100 (10 μM). Subsequently forskolin (1 μM) for 20 min was added and stimulation with CXCL12 (100 ng/ml) for 10 min was done. Controls include cells stimulated with CXCL12 and forskolin, or forskolin alone in absence of anti-CXCR4 inhibitors. Then the cells were harvested and lysed with 0.1 M HCl and cAMP levels was assayed using a direct competitive enzyme immunoassay (BioVision, Milpitas, CA, USA).

### Statistical analysis

Data are expressed as means ± SD of at least three independent experiments conducted in triplicates as indicated in the text and in the figure legends. Statistical significance was evaluated by *t-*test or Two-way ANOVA as indicated in the figure legends. Statistical significance was indicated as follows: *p* ≤ 0.05 (*), *p* ≤ 0.01 (**), *p* ≤ 0.001 (***) and *p* ≤ 0.0001 (****).

## Results

### Silencing of H ferritin triggers EMT in MCF-7 cells

**Fig. 1:**
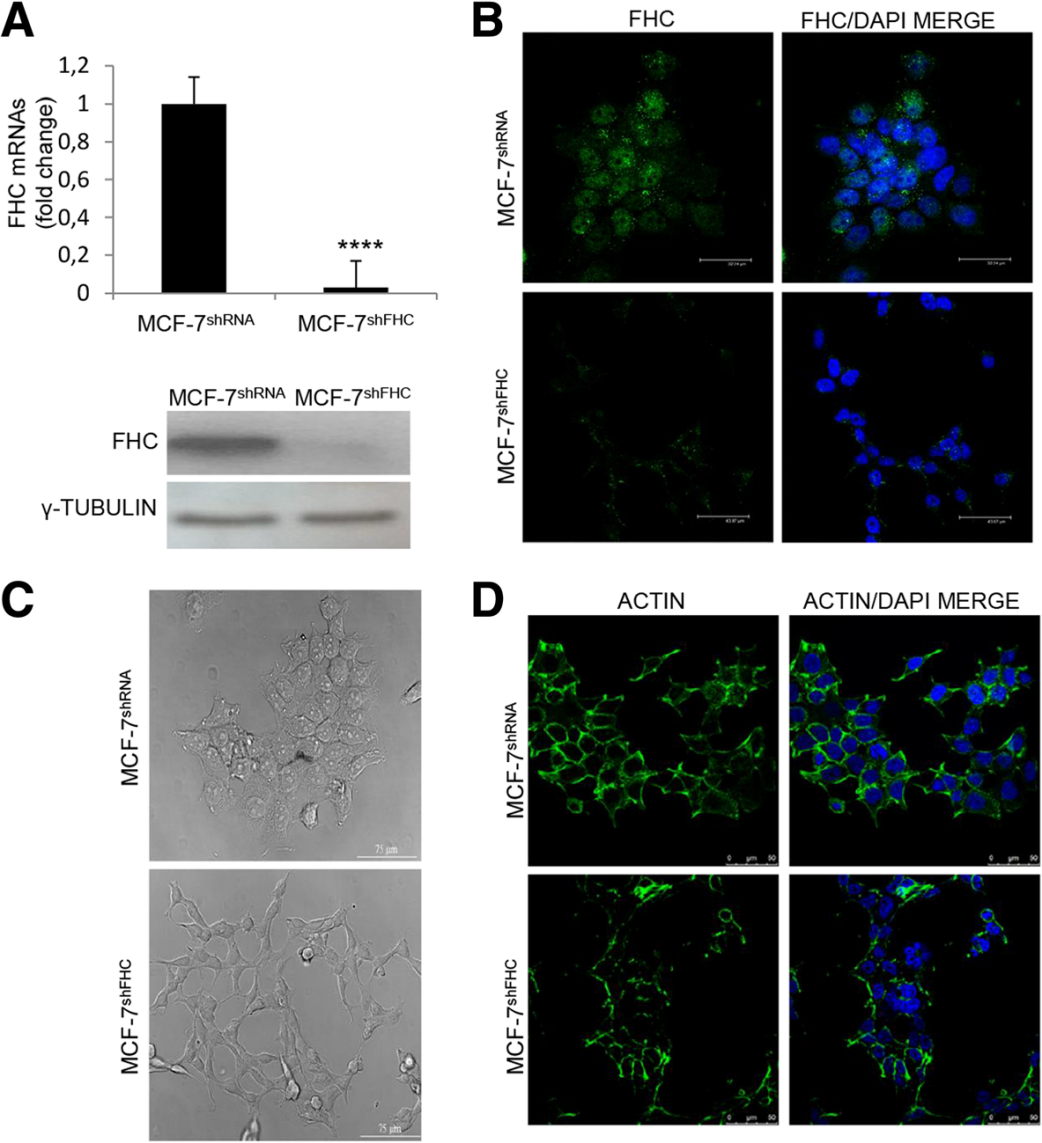
H ferritin gene silencing in MCF-7 cells. **a** Real-time PCR analysis of FHC mRNA expression was performed on total RNA extracted from MCF-7^shRNA^ and MCF-7^shFHC^ cells. Final results represent mean ± SD of three independent experiments. Statistical significance was evaluated by Student t-test (****, *p* < 0.0001). Western Blot analysis for FHC expression was performed on 50 μg of total protein extracts from MCF-7^shRNA^ and MCF-7^shFHC^ cells. γ-Tubulin was used as loading control. Representative data from one of three experiments. **b** MCF-7^shRNA^ and MCF-7^shFHC^ cells were fixed and incubated with monoclonal anti-FHC antibody (1:200) followed by incubation with the appropriate secondary antibody. Nuclei were visualized by DAPI staining. Images were collected using a Leica TCS SP2 confocal microscopy system (63X). Representative data from one of three experiments. **c** Images, collected using the phase-contrast microscope (Leica DMI 6000 CS), were used to highlight morphological changes between MCF-7^shRNA^ and MCF-7^shFHC^ cells. Representative data from one of three experiments. **d** MCF-7^shRNA^ and MCF-7^shFHC^ cells were fixed and incubated with Oregon Green 488 phalloidin (1:400) to visualize actin filaments. Nuclei were visualized by DAPI staining. Images were collected using confocal microscopy system (40X). Representative data from one of three experiments

We previously demonstrated that FHC intracellular amounts may regulate the expression of a number of miRNAs and EMT-related genes (miR-125b, Vimentin, and SPARC) in different cell types [17, 19, 34]. In this work, we evaluated the effects of FHC silencing on EMT in MCF-7 human breast cancer cells and in H460 human lung cancer cells. MCF-7 cells were stably transduced with a lentiviral DNA containing a Hferritin specific shRNA (MCF-7^shFHC^) or with an shRNA without significant homology to known human mRNAs (MCF-7^shRNA^). FHC mRNA and protein levels were measured in pools of silenced clones, as illustrated in Panel a of Fig. 1. Panel b shows a representative immunofluorescence for H ferritin subunit in MCF-7^shFHC^ and MCF-7^shRNA^ cells.

**Fig. 2:**
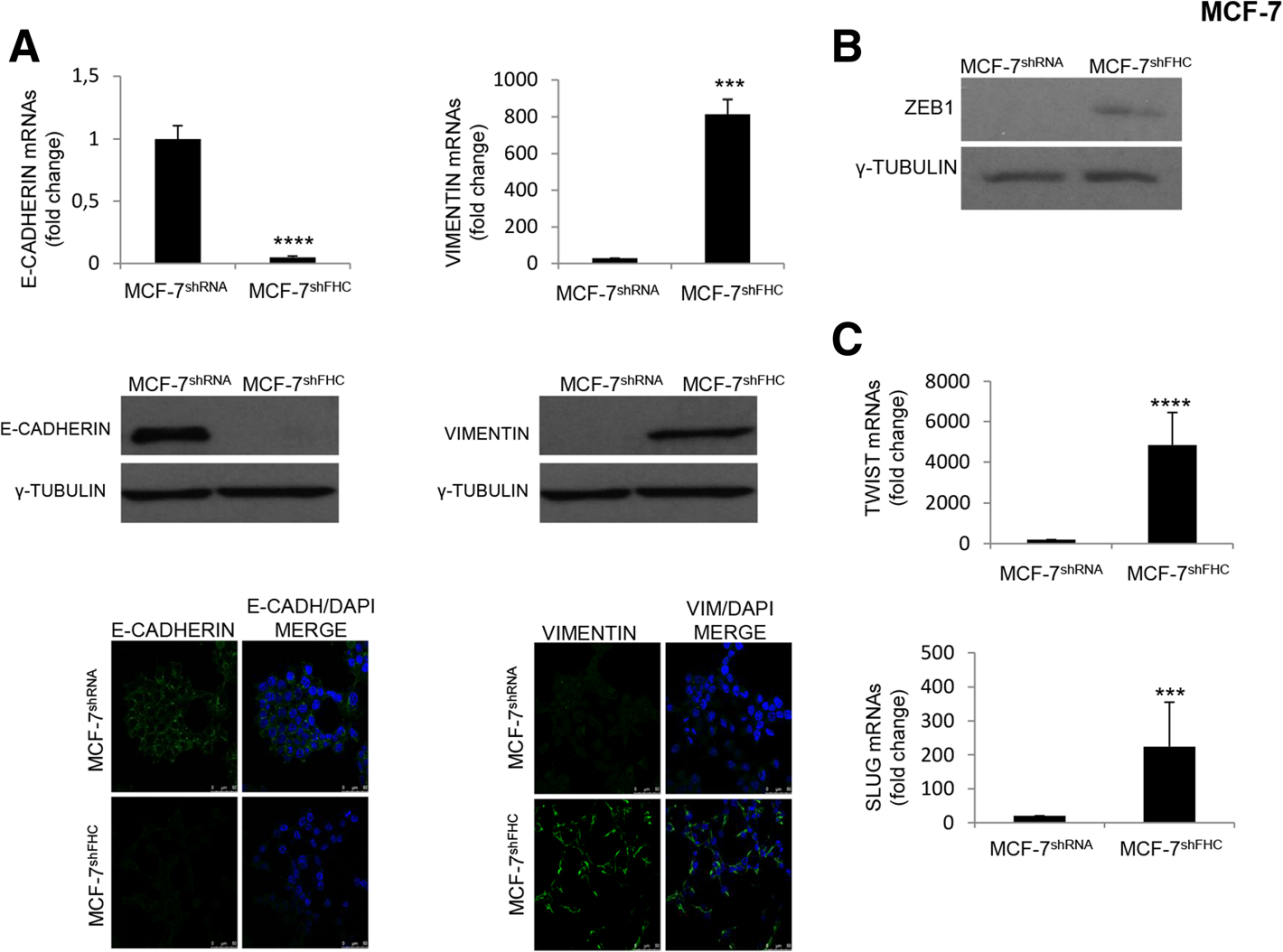
FHC-silencing and EMT gene expression in MCF7 cells. **a** E-Cadherin and Vimentin expression were evaluated by Real-time PCR, Western Blot and Immunofluorescence analysis. Real-time PCR were performed on total RNA extracted from MCF-7^shRNA^ and MCF-7^shFHC^ cells. Final results represent mean ± SD of three independent experiments. Statistical significance was evaluated by Student t-test (***, *p* < 0.001, ****, *p* < 0.0001). Western Blot analysis were performed on 50 μg of total protein extract from MCF-7^shRNA^ and MCF-7^shFHC^ cells. γ-Tubulin was used as loading control. For the Immunofluorescence analysis MCF-7^shRNA^ and MCF-7^shFHC^ cells were fixed and incubated with polyclonal anti-E-Cadherin or anti-Vimentin antibody (1:100) followed by incubation with the appropriate secondary antibody. Nuclei were visualized by DAPI staining. Images were collected using a Leica TCS SP2 confocal microscopy system (63X). Images are representative data from one of three experiments. **b** Western Blot analysis of ZEB1 was performed on 50 μg of total protein extract from MCF-7^shRNA^ and MCF-7^shFHC^ cells. γ-Tubulin was used as loading control. **c** Real-time PCR for Twist and Slug were performed on total RNA extracted from MCF-7^shRNA^ and MCF-7^shFHC^ cells. Final results represent mean ± SD of three independent experiments. Statistical significance was evaluated by Student t-test (**, *p* < 0.001)

FHC silencing produced dramatic morphological changes in MCF-7 cells. Indeed, phase-contrast microscope analysis revealed that the MCF-7^shFHC^ cells underwent a consistent modification, passing from a cobblestone-like morphology to a spindle-shaped and fusiform features (Panel C of Fig. 1), as well as cytoskeleton disarray, shown by staining of actin filaments with phalloidin (Panel D of Fig. 1). To corroborate the morphological data, we analysed the expression of several EMT markers in the MCF-7^shFHC^ and MCF-7^shRNA^ cells. Western blot, immunofluorescence and qPCR, reported in Fig. 2, show that FHC-silencing is accompanied by a decreased expression of E-cadherin, by an increase of Vimentin and by an up-regulation of EMT-related transcription factors Twist, Slug and ZEB1 [35–37].

**Fig. 3:**
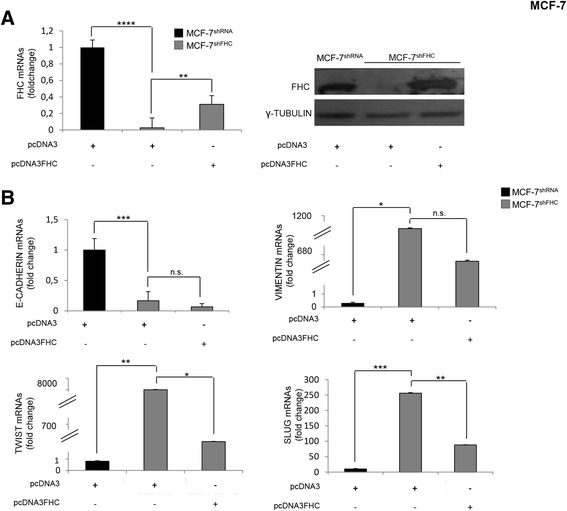
EMT markers and FHC amounts in MCF-7 cells. **a** Real-time PCR analysis of FHC was performed on total RNA extracted from MCF-7^shRNA/pcDNA^_3_, MCF-7^shFHC/pcDNA^_3_ and MCF-7^shFHC/pcDNA^_3_^FHC^ cells. Final results represent mean ± SD of three independent experiments. Statistical significance was evaluated by Student t-test (**, *p* < 0.01, ****, *p* < 0.0001). Western Blot analysis of FHC was performed on 50 μg of total protein extract from MCF-7^shRNA/pcDNA^_3_, MCF-7^shFHC/pcDNA^_3_ and MCF-7^shFHC/pcDNA^_3_^FHC^ cells. γ-Tubulin was used as loading control. Representative data from one of three experiments. **b** Real-time PCR analysis of E-Cadherin, Vimentin, Twist and Slug were performed on total RNA extracted from MCF-7^shRNA/pcDNA^_3_, MCF-7^shFHC/pcDNA^_3_ and MCF-7^shFHC/pcDNA^_3_^FHC^ cells. Final results represent mean ± SD of three independent experiments. Statistical significance was evaluated by Student t-test (*, *p* < 0.05, **, *p* < 0.01, ***, *p* < 0.001, n.s., not significant)

To strengthen the relationship between FHC down-regulation and EMT induction, we rescued FHC expression in MCF-7^shFHC^ cells (Fig. 3, Panel A), and re-evaluated the EMT markers. As shown in Panel B of Fig. 3, the expression of Vimentin, Twist and Slug was consistently reduced upon FHC reconstitution, while E-Cadherin levels were unaffected. We believe that this last phenomenon may be determined by the fact that the partial rescue of Twist and Slug in transiently FHC-reconstituted MCF-7 cells is still able to efficiently repress the expression of E-Cadherin. This hypothesis is confirmed by several published data reporting that the transcriptional regulation is indeed the major mechanism controlling E-Cadherin expression [35, 36].

### MCF7^shFHC^ cells display enhanced mobility, higher growth- and metabolic-rate

**Fig. 4:**
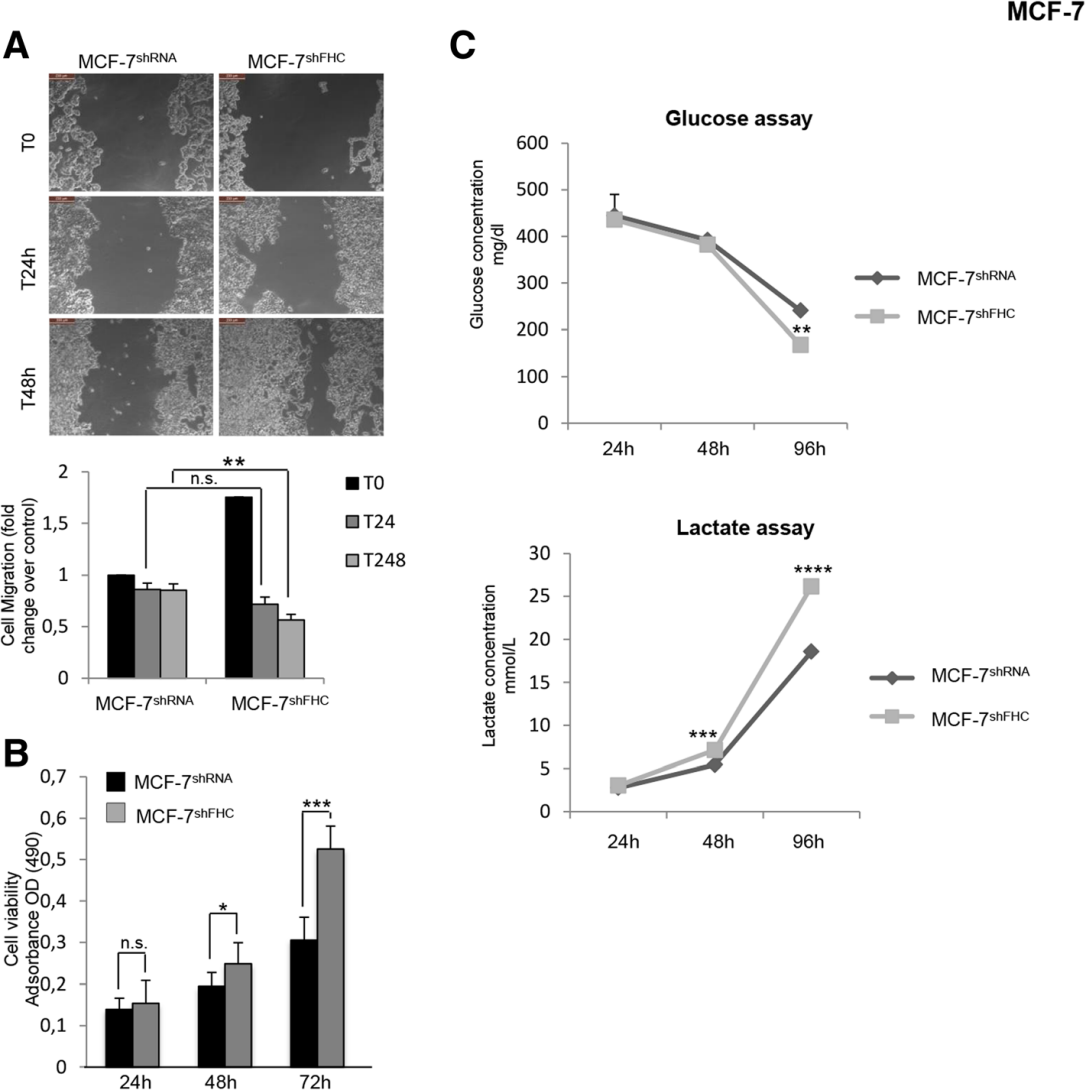
MCF-7^shFHC^ cells shows a more aggressive phenotype. **a** Wound healing assay was conducted to measure migration capacity of MCF-7^shRNA^ and MCF-7^shFHC^ cells. Images of cellular migration were taken at times 0 h, 24 h and 48 h (magnification of 10X) using the Leica DFC420 C and Leica Application Suite Software. Wound opening was quantified by ImageJ 64 software. Final results represent mean ± SD of three independent experiments. Cell migration fold change was evaluated using the T0 of MCF-7^shRNA^ as control. Statistical significance was evaluated by Two-Way ANOVA (Sidak’s) (n.s. not significant, **, *p*< 0.01). **b** Cell viability was assessed in MCF-7^shRNA^ and MCF-7^shFHC^ cells using the MTT method, as indicated in the Methods section, at 24 h, 48 h and 72 h. Final results represent mean ± SD of three independent experiments. Statistical significance was evaluated by Two-Way ANOVA (Sidak’s) (n.s. not significant, *, *p* < 0.01, ***, *p* < 0.001). **c** Cells were seeded in 10 ml of DMEM in 100 mm plates. After 24 h, 48 h and 96 h, 500 μl of supernatant were taken and glucose and lactate concentration was measured. Final results represent mean ± SD of three independent experiments. Statistical significance was evaluated by Two-Way ANOVA (Sidak’s) (**, *p* < 0.01, ***, *p* < 0.001, ****, *p* < 0.0001)

Epithelial cells undergoing EMT lose cell polarity and cell-to-cell adhesion capability, acquiring, in turn, enhanced migratory and invasive properties [37]. To further investigate the effects of FHC-silencing, we performed a wound healing assay on MCF-7^shFHC^ and control cells. The results of a triplicate set of independent assays (Panel A of Fig. 4) demonstrate that the MCF-7^shFHC^ cells possess, at 48 h, an enhanced migratory capability consistent with their EMT phenotype.

Next, we evaluated by methyl-thiazolyl-tetrazolium (MTT) assay the viability of FHC-silenced cells at 24, 48 and 72 h. The results, shown in Panel B of Fig. 4, indicate that FHC silencing increased cell proliferation with a maximum of about 1.8-fold at 72 h. Moreover, as further feature of cellular aggressiveness, the glucose/lactate levels were quantified over time in the MCF7^shFHC^ versus MCF7^shRNA^ cells. A time-dependent decrease in glucose concentration, paralleled by an increase in lactate levels were detected in the culture medium of MCF-7^shFHC^, indicating that the MCF-7^shFHC^ cells are indeed consuming higher amounts of glucose for energy production (Panel C of Fig. 4).

### The ROS scavenger N-acetylcysteine (NAC) partially rescued EMT in MCF-7^shFHC^

**Fig. 5:**
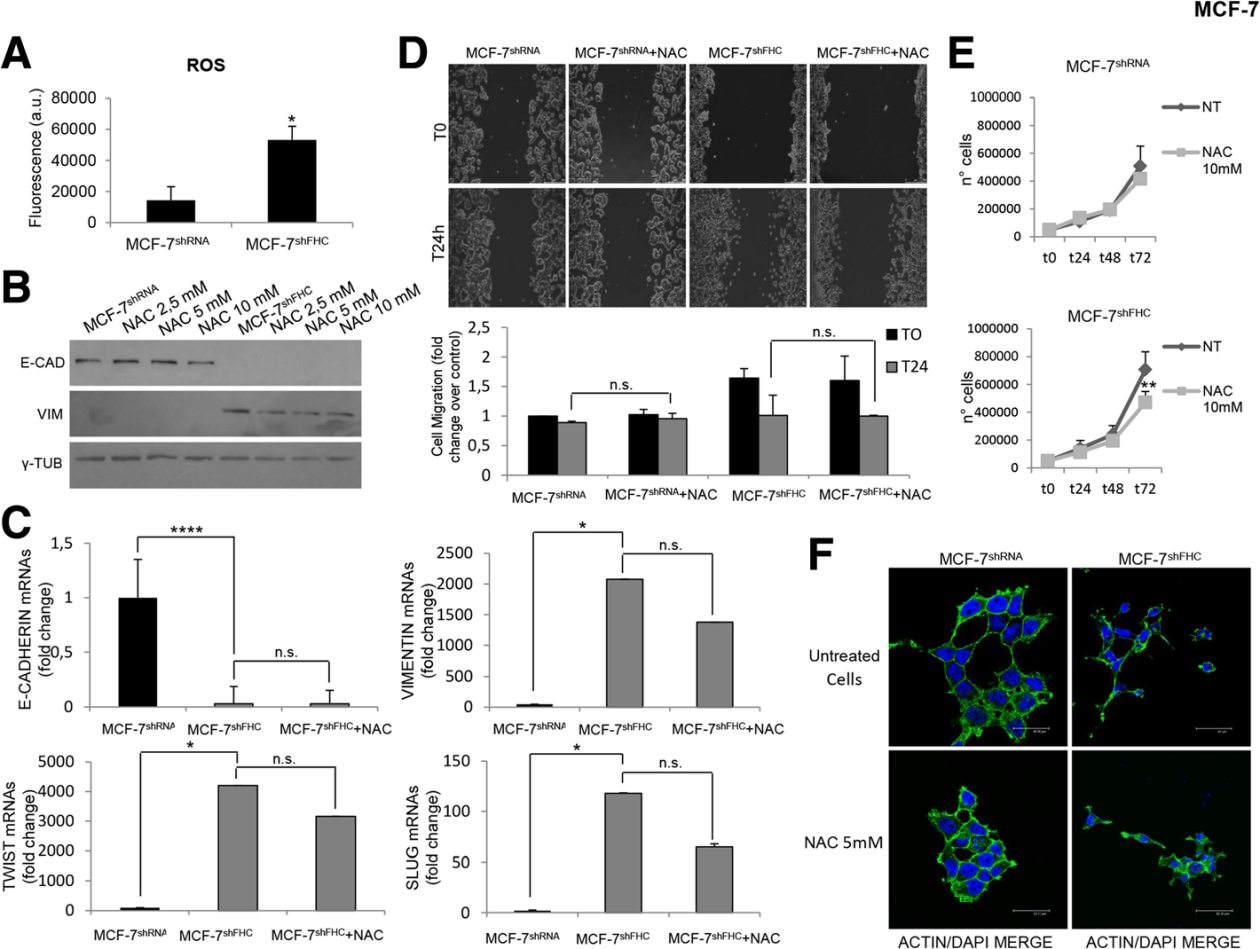
ROS levels and EMT in MCF-7^shFHC^ cells. **a** MCF-7^shRNA^ and MCF-7^shFHC^ cells (10^6^) were incubated for 15 min with 20 μM of 2′-7′-DCF and washed with HBSS solution. Fluorescence was measured using the FACS FORTESSA. Final results represent mean ± SD of three independent experiments. Statistical significance was evaluated by Student t-test (*, *p* < 0.05). **b** Western Blot analysis for E-Cadherin and Vimentin expression was performed on 50 μg of total protein extract from MCF-7^shRNA^ and MCF-7^shFHC^ cells treated with NAC (2,5–5-10 mM). γ-Tubulin was used as loading control. Representative data from one of three experiments. **c** Real-time PCR analysis of E-Cadherin, Vimentin, Twist and Slug mRNAs expression were performed on total RNA extracted from MCF-7^shRNA^, MCF-7^shFHC^ and MCF-7^shFHC^ cells treated with 5 mM NAC for 2 h. Final results represent mean ± SD of three independent experiments. Statistical significance was evaluated by Student t-test (*, *p* < 0.05, ****, *p* < 0.0001, n.s., not significant). **d** Wound healing assay was conducted to measure migration capacity of MCF-7^shRNA^ and MCF-7^shFHC^ cells treated and not treated with NAC (5 mM). Images of cellular migration were taken at times 0 h and 24 h (magnification of 10X) using The Leica DFC420 C and Leica Application Suite Software. Wound size was quantified by ImageJ 64 software. Final results represent mean ± SD of three independent experiments. Cell migration fold change was evaluated using the T0 of MCF-7^shRNA^ as control. Statistical significance was evaluated by Two-Way ANOVA (Sidak’s) (n.s., not significant). **e** Proliferation rate of MCF-7^shRNA^ and MCF-7^shFHC^ cells with NAC (10 mM) was assessed by cell count at 24, 48 and 72 h. Final results represent mean ± SD of three independent experiments each performed in triplicate. Statistical significance was evaluated by Two-Way ANOVA (Sidak’s) (**, *p* < 0.01). **f** MCF-7^shRNA^ and MCF-7^shFHC^ cells treated with NAC 5 mM were fixed and incubated with Oregon Green 488 phalloidin (1:400) to visualize actin filaments. Nuclei were visualized by DAPI staining. Images were collected using a Leica TCS SP2 confocal microscopy system (40X). Representative data from one of three experiments

Down-regulation of ferritin, and particularly of its heavy subunit, determines an increase of the intracellular Labile Iron Pool (LIP) [38–41]. The free iron, in turn, induces the formation of Reactive Oxygen Species (ROS), which are implicated in EMT induction in different cell types [10, 42, 43]. Panel A of Fig. 5 shows that, in MCF-7^shFHC^ cells, ROS amount is about five-fold greater than that in MCF-7^shRNA^ cells. To investigate to what extent increased ROS are related to EMT in our experimental model, we treated MCF-7^shFHC^ cells with the ROS scavenger N-acetylcysteine (NAC), and analyzed the levels of the EMT markers. NAC treatment partially reversed the expression of Twist (about 25%), Slug and Vimentin (about 40%), while it left unaffected the amounts of E-cadherin (Panels B and C of Fig. 5). The addition of NAC to the culture medium of MCF-7^shFHC^ cells did not substantially modify their migration capability (Panel D of Fig. 5), while, on the contrary, it significantly reduced their proliferation rate (Panel E of Fig. 5). Moreover, the spindle-shaped morphology of the silenced cells remained substantially unaltered upon NAC addition (Panel F of Fig. 5).

### CXCR4 axis is constitutively activated in MCF-7^shFHC^ cells

**Fig. 6:**
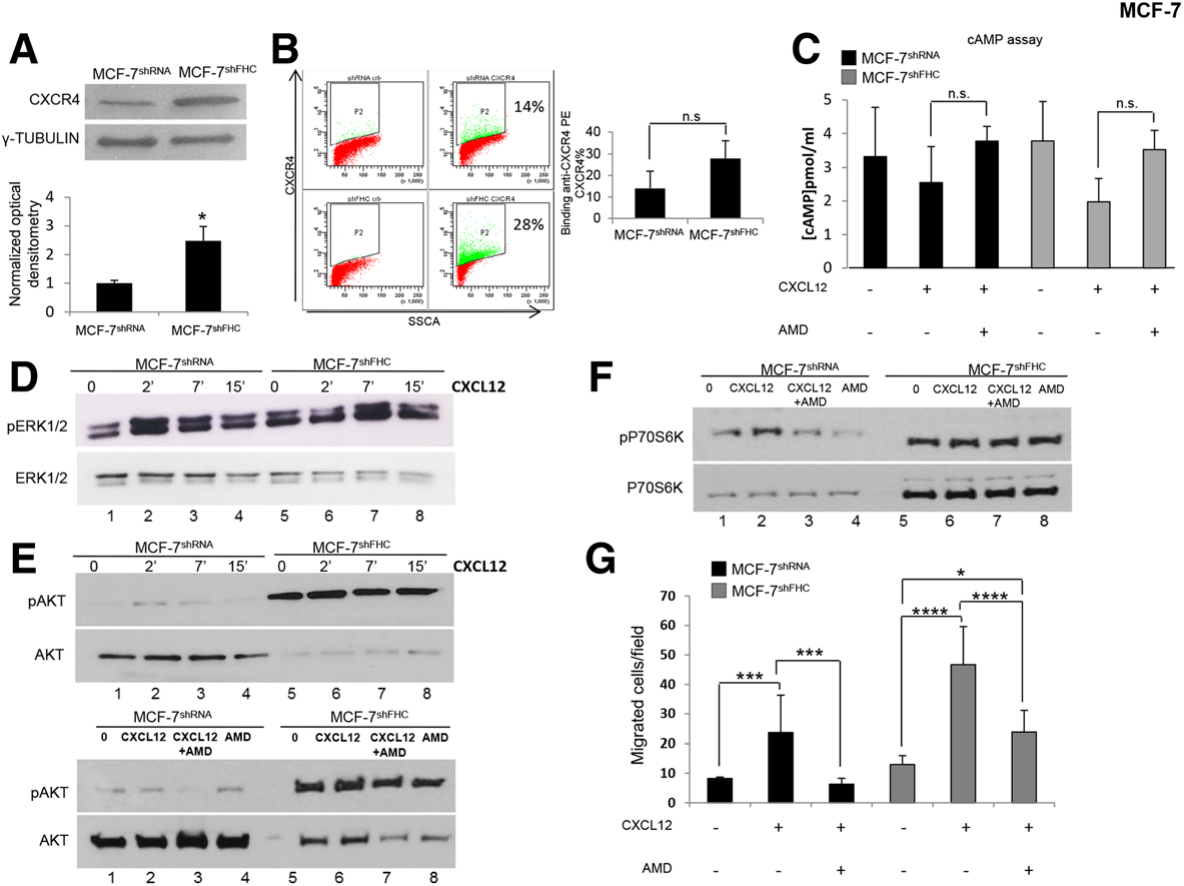
CXCR4 axis is activated in MCF-7^shFHC^ cells. **a** Western Blot analysis for CXCR4 expression was performed on 50 μg of total protein extract from MCF-7^shRNA^ and MCF-7^shFHC^ cells. γ-Tubulin was used as loading control. Representative data from one of three experiments. The graph represents the mean of the optical densities. Statistical significance was evaluated by Student t-test (*, *p* < 0.05). **b** Cells were stained with anti-CXCR4 PE (10 μL/1 × 10^6^ cells) and evaluated by a FACS Canto II cytofluorometer. Final results represent mean ± SD of three independent experiments. Statistical significance was evaluated by Student t-test (n.s., not significant). **c** cAMP production was verified after Forskolin (1 μM), CXCL12 (100 ng/mL), and AMD3100 (10 μM) treatment in MCF7 cells. Final results represent mean ± SD of three independent experiments. Statistical significance was evaluated by Two-Way ANOVA (Tukey’s) (n.s., not significant). **d** Western Blot analysis for pERK1/2 was performed on 50 μg of total protein extract from MCF-7^shRNA^ and MCF-7^shFHC^ cells. Cells were stimulated with CXCL12 (100 ng/ml) at the indicated time points. ERK1/2 was used as loading control. Representative data from one of three experiments. Numbers below the western blot indicates sample progression. **e Upper:** Western Blot analysis for pAKT was performed on 50 μg of total protein extract from MCF-7^shRNA^ and MCF-7^shFHC^ cells. Cells were stimulated with CXCL12 (100 ng/ml) at the indicated time points. AKT was used as loading control. Numbers below the western blot indicates sample progression. **Lower:** Cells were stimulated with CXCL12 (100 ng/ml for 10 min) and treated with the AMD3100 (10 μM) one hour before their exposure to CXCL12. AKT was used as loading control. Numbers below the western blot indicates sample progression. Representative data from one of three experiments. **f** Western Blot analysis for pP70S6 K expression was performed on 50 μg of total protein extract from MCF-7^shRNA^ and MCF-7^shFHC^ cells. Cells were stimulated with CXCL12 (100 ng/ml for 10 min) and treated with the AMD3100 (10 μM) one hour before their exposure to CXCL12. P70S6 K was used as loading control. Numbers below the western blot indicates sample progression. Representative data from one of three experiments. **g** CXCL12 dependent-cell migration was examined in 24-well plates. Cells were placed in the upper chamber (8 μm) in the presence of AMD3100 (10 μM). Cells migrated toward CXCL12 (100 ng/ml) for 18 h. The cells were counted in ten different consecutive high power fields (magnification 200×). Final results represent mean ± SD of three independent experiments. Statistical significance was evaluated by Two-Way ANOVA (Tukey’s) (*, *p* < 0.05, ***, *p* < 0.001, ****, *P* < 0.0001).

It has been shown that the binding of the chemokine CXCL12 to its CXCR4 receptor induces EMT in several cell types including MCF-7 cells [26–28, 44]. It has been also established that FHC binds the internalized CXCR4 receptor and impairs its transductional pathway [15] consisting, among others, in the inhibition of cAMP production and activation of ERK and AKT [45, 46]. In the subsequent experiments we investigated whether CXCR4 is activated in MCF-7^shFHC^ cells and to what extent this activation might play a role in the induction of the EMT phenotype. Upon FHC silencing, CXCR4 protein level increased of about two-fold, and the receptor was shifted towards the cell surface, even though not to a significant extent (Panels A and B of Fig. 6). In line with an activation of CXCR4 pathway in the silenced cells, the treatment with CXCL12 inhibited cAMP production more strongly in MCF-7^shFHC^ cells than in the control ones. Moreover, this effect was only partially rescued by the canonical CXCR4 inhibitor AMD3100 (Panel C of Fig. 6). We also evaluated the CXCR4-mediated activation of ERK1/2 and PI3K/Akt; the levels of phospho-ERK (pERK) were mildly increased in MCF-7^shFHC^ cells (Panel D of Fig. 6) and appeared substantially unaffected by treatment with CXCL12 and with AMD3100 (data not shown). Conversely, phospho-AKT (pAKT) was dramatically induced in MCF-7^shFHC^ (lanes 1 and 5 of upper panel E of Fig. 6) and the addition of CXCL12 did not further increase pAKT activity (lanes 2 and 6 of the same panel). The lower western blot in panel E of Fig. 6 shows that the addition of AMD3100 is unable to rescue the pAKT induction detected in MCF-7^shFHC^. Since CXCR4 has been shown to activate mTOR pathway via AKT [22] we also evaluated the levels of p-S6 ribosomal kinase (S6 K), the downstream effector of mTOR [47–49]. pAKT activation in MCF-7^shFHC^ cells corresponded to increased phosphorylation of S6 K, which is not inhibited by AMD3100 (Fig. 6 Panel F). Thus, the significant increase of pAKT and its downstream target S6 K, suggests a strong activation of survival pathways through CXCR4 following ferritin silencing.

To further explore the impact of CXCL12/CXCR4 axis activation on phenotypic changes produced by ferritin heavy chain silencing, we measured migration and proliferation in the presence of CXCL12 in MCF-7^shFHC^ versus MCF-7^shRNA^ cells. CXCL12 induces an approximately 3-fold increase of migration capability both in MCF-7^shRNA^ and MCF-7^shFHC^ cells. Interestingly, when MCF-7^shRNA^ and MCF-7^shFHC^ cells were exposed to AMD3100 a decrease in migration was detected (Panel G of Fig. 6). With regard to cell growth, the presence of CXCL12 in the culture medium did not produce any proliferative advantage in MCF-7^shFHC^ vs. MCF-7^shRNA^ and AMD3100 inhibited growth rate to the same extent both in FHC-silenced and unsilenced cells (data not shown).

### FHC silencing induces EMT and a more aggressive phenotype in H460 cells

**Fig. 7:**
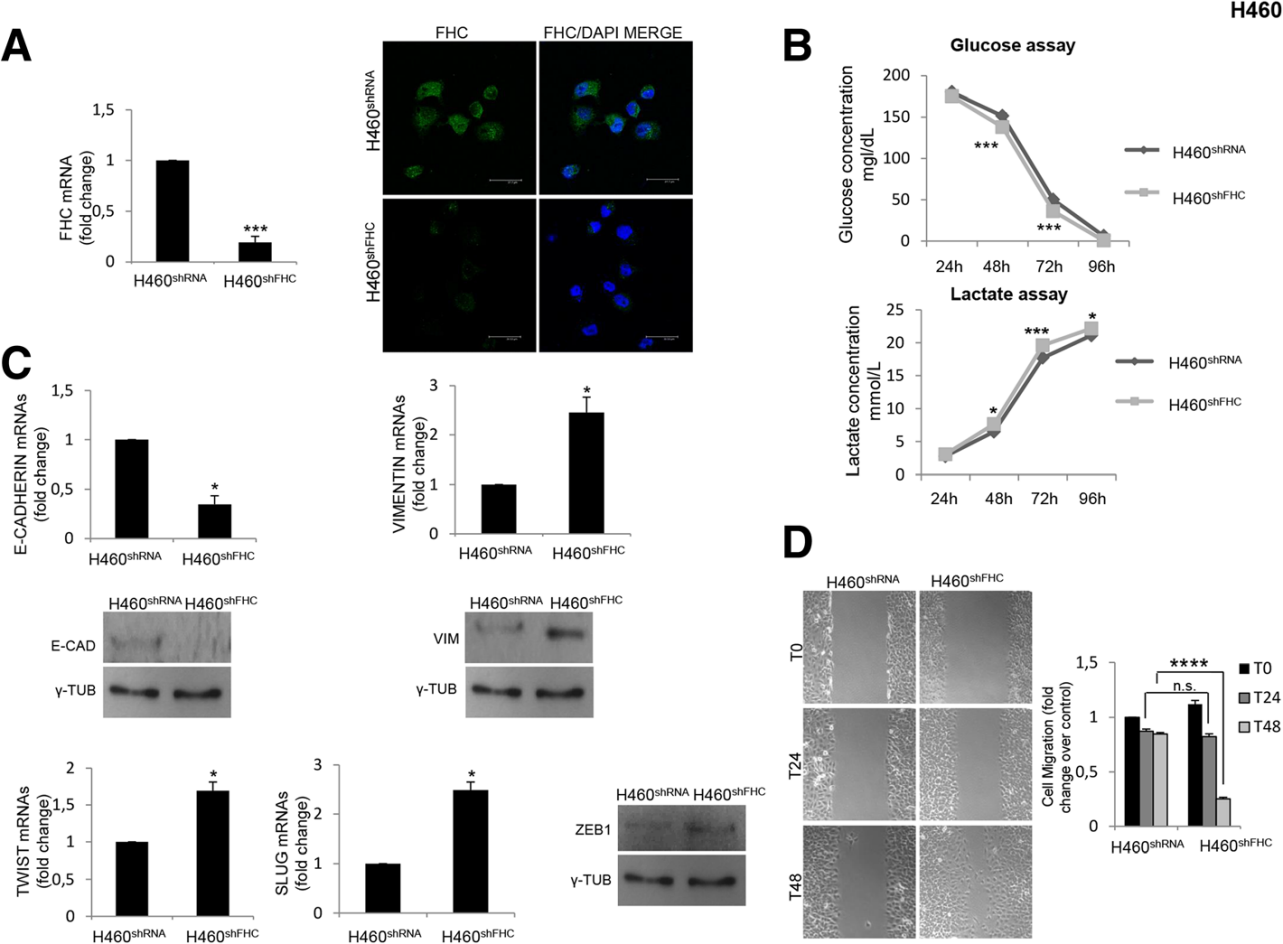
FHC silencing induces EMT in H460 cells. **a** Real-time PCR analysis of FHC mRNAs expression were performed on total RNA extracted from H460^shRNA^ and H460^shFHC^ cells. Final results represent mean ± SD of three independent experiments. Statistical significance was evaluated by Student t-test (***, *p* < 0.001). H460^shRNA^ and H460^shFHC^ cells were fixed and incubated with monoclonal anti-FHC antibody (1:200) followed by incubation with the appropriate secondary antibody. Nuclei were visualized by DAPI staining. Images were collected using a Leica TCS SP2 confocal microscopy system (63×). Representative data from one of three experiments. **b** Cells were seeded in 10 ml of RPMI in 100 mm plates. After 24 h, 48 h, 72 h and 96 h, 500 μl of supernatant were taken and glucose and lactate concentration was measured. Final results represent mean ± SD of three independent experiments. Statistical significance was evaluated by Two-Way ANOVA (Sidak’s) (*, *p* < 0.05, ***, *p* < 0.001). **c** Real-time PCR analysis of E-Cadherin, Vimentin, Twist and Slug. mRNAs expression was performed on total RNA extracted from H460^shRNA^ and H460^shFHC^ cells. Final results represent mean ± SD of three independent experiments. Statistical significance was evaluated by Student t-test (*, *p* < 0.05). Western Blot analysis for Vimentin, ZEB1 and E-Cadherin were performed on 100 μg of total protein extract from H460^shRNA^ and H460^shFHC^ cells. γ-Tubulin was used as loading control. **d** H460^shRNA^ and H460^shFHC^ cells migration was assessed using a wound-healing assay. Images of wounded monolayer of H460 cells were taken at times 0 h, 24 h and 48 h (magnification of 10X) using the Leica DFC420 C and Leica Application Suite Software. Wound size was quantified by ImageJ 64 software. Final results represent mean ± SD of three independent experiments. Cell migration fold change was evaluated using the T0 of H460^shRNA^ as control. Statistical significance was evaluated by Two-Way ANOVA (Sidak’s) (n.s., not significant ****, *p* < 0.0001)

To verify if the role of FHC silencing in EMT is restricted to MCF-7 cells or if it represents a more general phenomenon, we reproduced most of the analysis in the lung tumor NCI-H460 (H460) cells either FHC-silenced (H460^shFHC^) or unsilenced (H460^shRNA^) cells (Panel A of Fig.7). The reason for this choice lies in the fact that we recently published that FHC silencing of H460 cells determines increased ROS production, enhanced cell viability and activation of AKT [13]. Moreover, the analysis of glucose/lactate metabolism, shown in Panel B of Fig. 7, suggests a more aggressive phenotype of H460^shFHC^ compared to H460^shRNA^ cells. Consequently we evaluated the EMT markers by western blot and qPCR analysis. The results are shown in Panel C of Fig. 7.

**Fig. 8:**
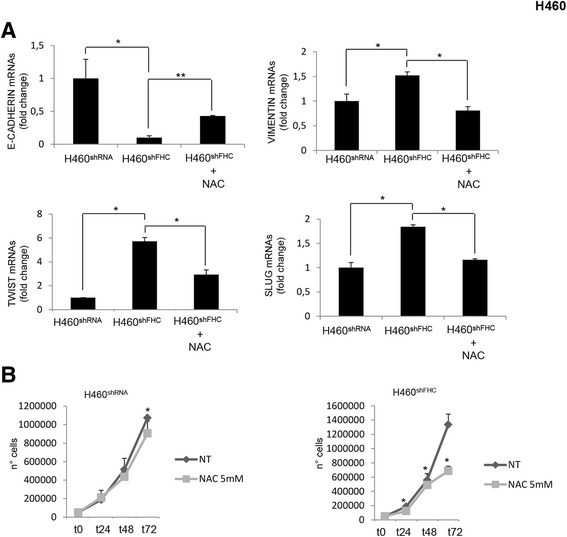
NAC treatment affects EMT markers expression and cell proliferation in H460 cells (**a**). Real-time PCR analysis of E-Cadherin, Vimentin, Twist and Slug mRNAs expression were performed on total RNA extracted from H460^shRNA^, H460^shFHC^ and H460^shFHC^ cells treated with 5 mM NAC for 2 h. Final results represent mean ± SD of three independent experiments. Statistical significance was evaluated by Student t-test (*, *p* < 0.05, **, *p* < 0.01). **b** Proliferation rate of H460^shRNA^ and H460^shFHC^ cells with NAC (10 mM) was assessed by cell count at 24, 48 and 72 h. Final results represent mean ± SD of three independent experiments each performed in triplicate. Statistical significance was evaluated by Two-Way ANOVA (Sidak’s) (*, *p* < 0.05)

As in MCF-7 cells, also in the H460 cells FHC knock-down is accompanied by a reduced expression of E-cadherin and by an up-regulation of Vimentin, Twist, Slug and ZEB1. Moreover, consistently with the acquisition of EMT properties, H460^shFHC^ cells gained greater migratory capacity than control cells at 48 h (Panel D of Fig. 7). The expression levels of these molecules were partially restored upon NAC treatment. Panel A of Fig. 8 shows that Twist levels are reduced of about 50% and that Slug and Vimentin are reduced of about 45% in the NAC-treated H460^shFHC^ compared to the untreated H460^shFHC^. Unlike MCF-7 cells, ROS chelation was able to partially restore E-cadherin expression. ROS chelation also reduced the enhanced proliferative rate of H460^shFHC^ cells, as shown in Panel B of Fig. 8.

**Fig. 9:**
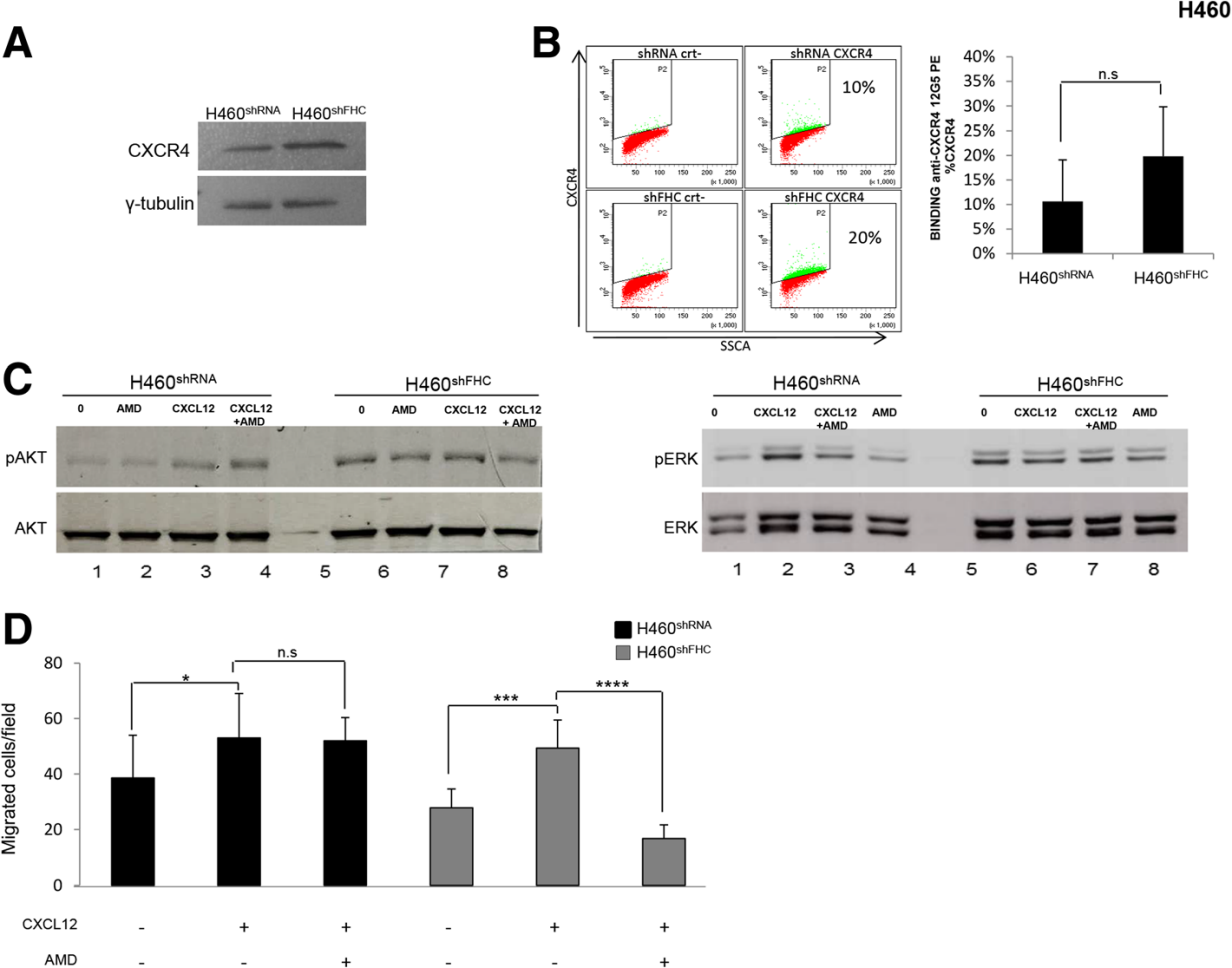
CXCR4 axis is activated in H460^shFHC^ cells. **a** Western Blot analysis for CXCR4 expression was performed on 50 μg of total protein extract from H460^shRNA^ and H460^shFHC^ cells. γ-Tubulin was used as loading control. Representative data from one of three experiments. **b** Cells were stained with anti-CXCR4 PE (10 μL/1 × 10^6^ cells) and evaluated by a FACS Canto II cytofluorometer. Final results represent mean ± SD of three independent experiments. Statistical significance was evaluated by Student t-test (n.s., not significant). **c** Western Blot analysis for pAKT and pERK expression was performed on 50 μg of total protein extract from H460^shRNA^ and H460^shFHC^ cells. Cells were stimulated with CXCL12 (100 ng/ml for 10 min) and treated with the AMD3100 (10 μM) one hour before their exposure to CXCL12. AKT and ERK were used as loading control. Numbers below the western blot indicates sample progression. Representative data from one of three experiments. **d** CXCL12 dependent-cell migration was examined in 24-well plates. Cells were placed in the upper chamber (8 μm) in the presence of AMD3100 (10 μM). Cells migrated toward CXCL12 (100 ng/ml) for 18 h. The cells were counted in ten different consecutive high power fields (magnification 200X). Final results represent mean ± SD of three independent experiments. Statistical significance was evaluated by Two-Way ANOVA (Tukey’s) (n.s., not significant, *, *p* < 0.05, ***, *p* < 0.001, ****, *P* < 0.0001)

As demonstrated in MCF-7 cells, FHC-silencing deregulated CXCR4 expression also in H460 cells. The steady-state amount of the receptor, evaluated by western blot, was about doubled in H460^shFHC^ cells with an increased localization, even though not statistically significant on the cell surface (Fig. 9, Panel a and Panel b). Along with the activation of AKT, which has been already demonstrated [13], FHC knock-down increased pERK expression, as shown in Panel C of Fig. 9. H460^shFHC^ cells were also treated with CXCL12 and with AMD3100 and pERK and pAKT levels were evaluated. The results, shown in Panel C of Fig. 9, are similar to those obtained with MCF-7^shFHC^ cells, since the levels of the two kinases were unaffected by treatment with CXCL12 and AMD3100. Finally, we analyzed the migration of H460 cells in the presence of AMD3100 demonstrating that the CXCR4 inhibitor consistently reduced the migratory capability of the H460^shFHC^ cells treated with CXCL12 (Panel D of Fig. 9).

Taken all together these results indicate that, albeit less dramatically than in MCF-7 cells, FHC silencing induces EMT and enhanced aggressiveness also in the lung tumor H460 cells and that these phenomena are attributable to both ROS increase and CXCR4 activation.

## Discussion

In the last years we and others have highlighted the role of ferritin heavy chain in the process of neoplastic transformation. We have documented that the modulation of FHC content determines, in different cell types, a severe alteration of gene expression, as evaluated by proteomic [29] and transcriptomic analysis [34]. In human metastatic melanoma cells this phenomenon is accompanied by a reduced invasiveness and adhesion along with a strong decrease in growth activity [29]. On the contrary, in two epithelium-derived cell lines, namely the human lung cancer cells H460 and the ovarian cancer cells SKOV3, FHC-silencing induced an increase of cell proliferation rate [13, 19]. In addition, in SKOV3 cells FHC knock-down led to the acquisition of stemness properties along with a strong EMT phenotype [19]. Other groups have highlighted the role of FHC in neoplastic cells, underlining, for example, its ability to modulate the activity of p53 and DAXX [14, 50]. The molecular basis of this phenomenon is still under investigation and conflicting results have been obtained. Zhang et al. demonstrated that the acquisition of EMT phenotype in AML-12 murine hepatocytes and in human A459 lung cancer cells is due to increased ROS production induced by FHC down-regulation [10]. In SKOV3 cells we correlated the EMT and the more aggressive phenotype of FHC-silenced cells to the altered expression of miR-125b, miR-146a and miR-150 [19].

Here, we studied the role of FHC in MCF-7 human breast cancer cells and in H460 lung cancer cells, demonstrating that silencing of ferritin heavy subunit increases cell aggressiveness through induction of EMT, increased proliferation rate and migratory ability. The major finding of this work is that FHC knock-down is accompanied, along with increased ROS production, by a strong activation of the CXCR4 transductional pathway, and that both these phenomena are responsible for the enhanced tumorigenic phenotype. In both MCF-7 and H460 cells, indeed, the treatment of the FHC-lacking cells with the ROS inhibitor NAC does not fully rescue the EMT phenotype while significantly affects the proliferation rate of silenced cells. On the contrary, MCF-7^shFHC^ and H460^shFHC^ cells show a strong reduction of their migratory capability when exposed to the CXCR4 inhibitor AMD3100.

These results are in line with the actual concept that FHC is a multifunctional molecule involved, from one hand, in intracellular iron homeostasis, and from the other in pathways not directly related to iron metabolism, such as the inhibition of cell death [13, 51, 52]. Among the non-iron related functions, FHC is able to interfere with CXCR4 signalling. Previous evidences have shown that FHC binds and inhibits CXCR4 function in HEK293 and in HeLa cells; the CXCR4-mediated ERK1/2 activation and chemotaxis are strongly inhibited by FHC-overexpression and, conversely, prolonged by FHC down-regulation [15]. Moreover, the binding of FHC to CXCR4 induces FHC phosphorylation at serine 178 and nuclear translocation [15]. The functional outcomes of CXCR4/FHC interaction have been widely investigated in neurons, in both physiological and pathological conditions [53–55]. In these cells, the long-term treatment with Mu opioid receptor (MOR) agonists, such as morphine, inhibits CXCL12-induced activation of CXCR4 and the related downstream signalling through ERK and AKT [56]. The opiate-induced inhibition of CXCR4 requires de novo protein synthesis and up-regulation of FHC that acts as negative modulator of CXCR4 [15]. The neuronal levels of FHC are augmented by MOR stimulation both in vitro (i.e. neuronal cultures) and in vivo (i.e. rat brain), always leading to CXCR4 impairment [56].

Our data indicate that in MCF-7 and H460 cells FHC knock-down increases CXCR4 surface expression and signalling, and that the activation of this pathway is largely responsible for the increase in migration capability. The analysis of the CXCR4 downstream patterns shows a consistent analogy with the reported role of FHC in neurons, since also in MCF-7 and H460 cells FHC down-regulation is accompanied by activation of ERK and AKT. Interestingly, pERK and pAKT amounts in silenced cells are not further modified by CXCL12 treatment or by the use of the CXCR4 inhibitor AMD3100, while the inhibition of cAMP production is responsive to the cytokine as well as to the inhibitor. The unresponsiveness of pAKT to CXCL12 and AMD3100 is likely due to the fact that the silencing of ferritin induces a constitutive active CXCR4 transduction pathway, which is poorly affected by the two treatments. Overall, these data suggest that, among the downstream targets of CXCR4, FHC insists particularly on the signalling mediated by MAP Kinases and PI3K-pAKT pathways. Moreover, the increase in pAKT expression levels in MCF-7^shFHC^ cells corresponds to S6 K induction suggesting an activation of the AKT-mTOR-related survival pathway, as previously reported downstream of CXCR4 in neurons and renal cell lines [22].

CXCR4 and its downstream pathways have been widely investigated in cancer and EMT. Among others, Roccaro et al. [55] demonstrated that CXCR4 enhances the acquisition of an EMT-like phenotype in multiple myeloma (MM) cells with a phenotypic conversion towards invasion, leading to higher bone metastasis and extramedullary disease dissemination in vivo. In contrast, CXCR4 silencing leads to inhibition of tumor growth and to reduced survival. Grundker et al. [44] showed that CXCL12 induces invasion and EMT genes in MCF-7 and T-47-D breast cancer cells. Less is known about the interference of FHC on CXCR4 signalling in neoplastic cells; to our knowledge this is one of the first reports on the CXCR4-mediated FHC role in cancer.

The differences between our findings in MCF-7 and H460 cells and those of Zhang et al. in AML-12 and A459 cells deserve a further consideration. In AML-12 cells, in fact, EMT is exclusively promoted by ROS increase, which, in turn, is induced by FHC down-regulation. ΝΑC treatment completely reversed EMT phenotype and strongly affected proliferation and migration indexes. We hypothesize that the different behaviour of FHC-silencing might be attributed to a different cell-specific activity of the ferritin heavy subunit. In the context of the emerging multiple functions played by this molecule, it is reasonable to postulate that FHC acts as house-keeping protein for the functions related to iron/redox metabolism, while its role in other metabolisms is strictly dependent on the cell type.

## Conclusions

Despite the large number of studies on ferritin and particularly on its heavy subunit in neoplastic cells, the role of FHC in CXCR4 signalling in cancer has not been addressed so far. The activation of CXCL12/CXCR4 axis enhances migration and invasion of cancer cells, thus leading to metastasis [57, 58] and may regulate antitumor immune response through T cells access [59]. Thus, the integrity of FHC-regulated pathways may be a tool to predict tumor aggressiveness. Increased EMT, migration and survival depend on release of FHC control on several pathways such as ROS and CXCR4; therefore, targeting FHC-ROS-CXCR4 axis could reverse these effects. Further studies are needed to define in details the molecular effects of FHC/CXCR4 interaction; nevertheless, we believe that our findings might have relevant implications in the design of new therapeutic approaches.

## Abbreviations

BSA: Bovine serum albumin
CNS: Central nervous system
CXCL12: C-X-C motif chemokine ligand 12
CXCR4: C-X-C chemokine receptor type 4
DMEM: (Dulbecco’s Modified Eagle Medium
ECL: Electrochemiluminescence
EMT: Epithelial to mesenchymal transition
FBS: Fetal bovine serum
FHC: Heavy chain of ferritin
FTH or FHC: Ferritin heavy chain
FTL: Ferritin light chain
GAPDH: Glyceraldehyde 3-phosphate dehydrogenase
GPCR: G protein-coupled receptor
HEK-293 T: Human embryonic kidney cells 293
HS: High salt solution
LIP: Labile Iron Pool
MOR: Mu opioid receptor
MTT: 3-(4,5-dimethylthiazol-2-yl)-2, 5-diphenyl tetrazolium bromide
NAC: *N-*acetylcysteine
O.D: Opticla Density
PBS: Phosphate buffered saline
PFA: Paraformaldehyde
qRT-PCR: Quantitative RT-PCR
ROS: reactive species of oxygen
RPMI: Roswell Park Memorial Institute medium
SDS: Sodium dodecyl sulfate
shRNA: Short hairpin
TPBS: Tris buffered saline
